# Scedosporium infection as a late complication after start of elexacaftor/tezacaftor/ivacaftor: two case reports

**DOI:** 10.1016/j.rmcr.2025.102278

**Published:** 2025-08-30

**Authors:** P van Mourik, E.C. van der Hout, H.D. Luijk, R.W. Hofland, A.H.W. Bruns, H.G.M. Heijerman, I. Bronsveld

**Affiliations:** a Department of Pulmonary Medicine, University Medical Center Utrecht, the Netherlands; b Department of Reumatology,immunology and infectious diseases, University Medical Center Utrecht, the Netherlands

**Keywords:** Scedosporium, Cystic fibrosis, Elexacaftor/tezacaftor/ivacaftor

## Abstract

Highly effective modulator treatment (HEMT) has resulted in an improved prognosis for people with cystic fibrosis (pwCF). In this case report, we present two pwCF with clinical stabilization after start of HEMT who developed complicated fungal infections after several years of clinical stability, highlighting the importance of continued careful monitoring of pwCF on HEMT and considering early antifungal treatment in this population.

## 1 Introduction

The advent of highly effective Cystic Fibrosis Transmembrane conductance Regulator (CFTR)-modulator therapy (HEMT) for people with Cystic Fibrosis (pwCF) has dramatically improved the lives and prognosis of eligible pwCF [1,2]. Importantly, bacterial load of well-known pathogens such as pseudomonas aeruginosa has been shown to decrease [3], thereby removing an important contributor to disease exacerbations. Moreover, in pwCF with severe disease, a reduction of the need for lung transplantation has been observed [4]. With these significant improvements in disease severity, a decrease in check-up frequency would seem logical.

Nevertheless, structural parenchymal damage often persists after clearance of sputum and bacterial colonization. These architectural distortions provide a niche for other opportunistic pathogens such as fungi. Fungal colonization is common in CF, but the clinical relevance remains uncertain and might be different in pwCF on HEMT. Here, we present two pwCF with initial clinical stabilization after start of HEMT that experienced progressive fungal infections, highlighting the importance of continued imaging and monitoring of sputum cultures and considering treatment of pwCF using CFTR-modulators.

## 2 Case presentation

Both patients provided informed consent for publication of this case report.

Our first case is a 41-year old male with Cystic Fibrosis homozygous for F508del, who started with lumacaftor/ivacaftor in 2018, but his FEV1 continued to decline during treatment (see Table 1 for timeline of relevant events). During 2018 and 2019 he was extensively treated with different antibiotics, caspofungin and voriconazole with only short-term improvement. Therefore, he was screened for lung transplantation in 2019. Because of his fast clinical deterioration, elexacaftor/tezacaftor/ivacaftor (ETI) was started on a compassionate use basis in February 2020. After starting ETI his FEV1 improved from 23 percent predicted (pp) to 32 pp, his exacerbation frequency decreased significantly until 2022 and he was taken off the candidate list for lung transplantation.

**Table 1:** Timeline Case 1 (41-year old male).

Year	Event	Fungal therapy?
2005	First sputum culture with scedosporium species	No
2018	Start lumacaftor/ivacaftor	No
2018	First computed-tomography scan with cavitary lesions	No
2019	Frequent exacerbations and hemoptysis not responding to antibiotics	Caspofungin, voriconazole
2020–02	Start of elexacaftor/tezacaftor/ivacaftor	No
2020–06	Hemoptysis	Voriconazole
2022–08	Recurrence of exacerbations	Voriconazole
2022–12	Persistent exacerbation	Voriconazole oral
		Amphotericine inhalation
2023–06	Persistently subtherapeutic voriconazole serum levels and invalidating ocular side-effects	Olorofim
2023–12	Secondary pneumothorax, pleural infection with scedosporium	Olorofim
		Voriconazole
		Intrapleural olorofim
		Intrapleural voriconazole

**Fig. 1:**
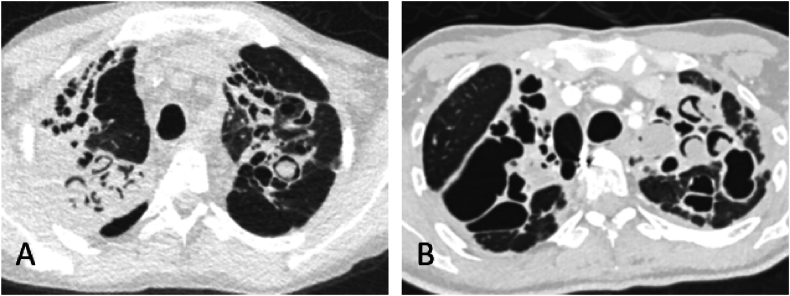
Computed Tomography images of case 1 (A) and case 2 pre-transplantation (B). In both images, severe bronchiectasis with rounded masses inside cavities with the air crescent sign can be seen, typical of fungal infection.

Unfortunately in 2022 frequent exacerbations and hemoptysis reoccurred, with limited response to antibiotics. The patient was colonized with Aspergillus Fumigatus and Scedosporium species since 2005. In retrospect there was no evidence of aspergilloma, allergic bronchopulmonary aspergillosis or chronic pulmonary aspergillosis until 2018. In 2018, the first indications of an aspergilloma were seen on a CT-thorax (Fig. 1). Over the years, the amount and size of caverns with a rounded mass increased and he experienced frequent hemoptysis. Fungal infection was suspected to be the cause of his detoriation and voriconazole and later inhaled amphotericin B were (re)started end of 2022, almost three years after start of ETI. Unfortunately, he developed severe photosensitivity with voriconazole and his serum levels were below therapeutic range. Therefore, the investigational antifungal agent olorofim [5] was started on compassionate use basis in June 2023. Subsequently, he stopped experiencing hemoptysis. In December 2023, he developed a secondary pneumothorax with persistent air leakage. This was thought to be due to structural lung damage, and not necessarily progression of fungal infection. Pleural fluid cultures showed scedosporium species attributed to spillage after pneumothorax. After long-term pleural drainage, twice daily intrapleural washing with both voriconazole and compassionate use olorofim and reintroducing voriconazole orally, his drain could be removed and he could be discharged home. However, because of progressive pulmonary decline he has been reinstated on the waiting list for lung transplantation.

The second case is a 38-year old female with Cystic Fibrosis (F508del/R553X), who was on the waiting list for lung transplantation since 2014, and had started ETI in a compassionate use program in 2019 because of progressive decline of pulmonary function (see Table 2 for timeline of relevant events). Subsequently, her FEV1 improved from 34pp to 40pp, was taken off the waiting list for lung transplantation, and she stopped experiencing pulmonary exacerbations until 2021, when she was hospitalized with an ongoing pulmonary exacerbation and recurring hemoptysis for which embolization was required. The patient was treated with intravenous antibiotics for several months, with limited effect. Scedosporium species had been cultured in her sputum multiple times since 2014 and therefore fungal infection was suspected to contribute to her ongoing exacerbation, especially because her CT-scans since 2019 indicated the presence of fungi in her bronchiectasis (Fig. 1). Voriconazole was added to her treatment. After start of voriconazole, her hemoptysis disappeared. She was replaced on the waiting list for lung transplantation because of a permanent need for meropenem and voriconazole, and was successfully transplanted in 2023. In the explanted lungs, extensive hyphae were found consistent with fungal infection. Unfortunately, post-transplantation pleural fluid contained Scedosporium species, consistent with spill during transplantation, for which long-term voriconazole was started.

**Table 2:** Timeline case 2 (38-year old female).

Year	Event	Fungal therapy?
2011	First sputum culture with scedosporium species	No
2019	First computed-tomography scan with cavitary lesions	No
2019	Start elexacaftor/tezacaftor/ivacaftor	No
2022	Recurrence of exacerbation	No
2022–09	Persistent exacerbation	Voriconazole
2023–09	Lung transplantation, post-transplant pleural effusion contaminated with scedosporium	Voriconazole

## 3 Discussion

These described cases emphasize the importance of awareness of clinically relevant fungal infections in all pwCF, especially when taking ETI. Both cases showed initial stabilization and improvement in pulmonary function, and both were taken off the lung transplantation list. However, after several years, disease exacerbations returned, attributed to fungal infections. Long-term data after start of ETI is limited to several years, and therefore limited data is available about possible fungal complications. These cases highlight that parenchymal lung damage creates a niche for opportunistic pathogens despite improved mucociliary clearance after start of ETI.

Many pwCF are colonized with fungi, and the clinical relevance of Scedosporium species is not fully known, although colonization is associated with increased exacerbation rates [6]. Moreover, clinically differentiating between bacterial exacerbation and fungal infection is difficult. Biomarkers such as Aspergillus Fumigatus-specific serum IgG is a sensitive marker for pulmonary aspergillosis. However, serologic testing is not available for Scedosporium species. Some reviews suggest imaging could be helpful to distinguish fungal colonization from infection [7]. In our cases, evidence of aspergillomas or possibly Scedosporium balls (‘Scedosporiomas’) was clearly present on CT-scans. Therefore, frequent imaging could potentially be used to monitor for progressive fungal infection in patients that are persistently colonized with fungi.

Voriconazole is considered first line treatment for Scedosporium species, although some suggest combined therapy could be more effective [8]. In our cases, voriconazole at therapeutic dose was initially effective at treating or at least suppressing infection. However, subtherapeutic triazole concentrations have been reported in combination with CFTR modulators, which was also problematic in our first case [9]. Moreover, many questions remain, such as the optimal treatment duration, whether inhaled antifungals could have a role, and what the clinical endpoints for efficacy should be. Interestingly, both cases presented with recurrent hemoptysis which resolved after start of antifungal treatment. Therefore, prompt treatment with anti-fungal agents in pwCF who have evidence of pulmonary scedosporiosis and recurrent hemoptysis should be considered.

In pwCF with HEMT, the role of fungal colonization and possible effects of antifungal treatment on long-term outcomes have not been definitively established. However, our cases highlight the risk of fungal infection including Scedosporium species in this patient group. Whether the decreased bacterial load and improved mucociliary clearance after HEMT create a more advantageous environment for fungi needs further exploration. The interplay between bacteria and fungi is complex, with conflicting literature suggesting a possible inhibitory effect of pseudomonas on aspergillus [10]. If pseudomonas bacterial load decreases, fungi might take a more central role in pulmonary deterioration in pwCF on HEMT. Our cases stress the necessity of regular follow-up regardless of CFTR-modulator use, and raise the question whether more frequent imaging and aggressive treatment of fungi after start of HEMT should be considered.

## CRediT authorship contribution statement

**P van Mourik:** Writing – review & editing, Writing – original draft, Investigation, Data curation, Conceptualization. **E.C. van der Hout:** Writing – review & editing, Conceptualization. **H.D. Luijk:** Writing – review & editing. **R.W. Hofland:** Writing – review & editing, Data curation. **A.H.W. Bruns:** Writing – review & editing. **H.G.M. Heijerman:** Writing – review & editing, Conceptualization. **I. Bronsveld:** Writing – review & editing, Conceptualization.

## Declaration of competing interest

The authors declare the following financial interests/personal relationships which may be considered as potential competing interests: HGMH reports speaker fees from 10.13039/100019719Chiesi, 10.13039/100012754Horizon Pharma, 10.13039/100013223PTC Therapeutics, TEVA, and Vertex; and fees for advisory board participation from Vertex and 10.13039/100013223PTC Therapeutics.
